# Modulating Both Tumor Cell Death and Innate Immunity Is Essential for Improving Radiation Therapy Effectiveness

**DOI:** 10.3389/fimmu.2017.00613

**Published:** 2017-05-26

**Authors:** Qiuji Wu, Awatef Allouch, Isabelle Martins, Catherine Brenner, Nazanine Modjtahedi, Eric Deutsch, Jean-Luc Perfettini

**Affiliations:** ^1^Cell Death and Aging Team, Gustave Roussy Cancer Campus, Villejuif, France; ^2^Laboratory of Molecular Radiotherapy, INSERM U1030, Gustave Roussy Cancer Campus, Villejuif, France; ^3^Gustave Roussy Cancer Campus, Villejuif, France; ^4^Université Paris Saclay, Villejuif, France; ^5^Department of Radiation and Medical Oncology, Zhongnan Hospital, Wuhan University, Wuhan, China; ^6^Hubei Key Laboratory of Tumor Biological Behaviors, Zhongnan Hospital, Wuhan University, Wuhan, China; ^7^Laboratory of Signaling and Cardiovascular Pathophysiology, INSERM UMR-S 1180, Université Paris-Sud, Faculté de Pharmacie, Châtenay-Malabry, France

**Keywords:** ionizing radiation, tumor cell death, innate immunity, immunotherapy, cancer treatment

## Abstract

Radiation therapy is one of the cornerstones of cancer treatment. In tumor cells, exposure to ionizing radiation (IR) provokes DNA damages that trigger various forms of cell death such as apoptosis, necrosis, autophagic cell death, and mitotic catastrophe. IR can also induce cellular senescence that could serve as an additional antitumor barrier in a context-dependent manner. Moreover, accumulating evidence has demonstrated that IR interacts profoundly with tumor-infiltrating immune cells, which cooperatively drive treatment outcomes. Recent preclinical and clinical successes due to the combination of radiation therapy and immune checkpoint blockade have underscored the need for a better understanding of the interplay between radiation therapy and the immune system. In this review, we will present an overview of cell death modalities induced by IR, summarize the immunogenic properties of irradiated cancer cells, and discuss the biological consequences of IR on innate immune cell functions, with a particular attention on dendritic cells, macrophages, and NK cells. Finally, we will discuss their potential applications in cancer treatment.

## Introduction

Radiation therapy has been used in cancer treatment for over a century and represents one of the most efficient treatment modalities in the oncology field. Over 50% of all cancer patients receive radiation therapy during the course of their disease. Radiation therapy is widely used in many localized solid tumors, ranging from brain tumors, head and neck cancer, lung cancer, esophageal cancer, breast cancer, rectal cancer, and cervical cancer to prostate cancer among others. Radiation therapy is also used for the management of metastatic diseases such as brain or bone metastasis (1). Despite the fact that radiation therapy contributes to approximately 40% of all cancer cures (2), treatment failure is frequently observed due to local recurrence and distal metastasis (3).

Antitumor effects of radiation therapy are mainly due to the induction of an important cellular stress that triggers cell cycle arrest and leads eventually to either cellular senescence or cell death depending on the doses and the irradiation schedules used. Today it is also established that these local biological effects stimulate both innate and adaptive immune cells present in the tumor microenvironment and elicit an antitumor response at distance of the irradiated tumor sites. This biological process is also known as “abscopal” effect. The antitumor response elicited by radiation therapy can be enhanced by unleashing immune resistance mechanisms through the use of immune checkpoint blockers [such as anti-cytotoxic T-lymphocyte-associated protein-4 (anti-CTLA-4) or anti-PD-L1 antibodies], revealing that the modulation of the cross-talk between the biological effects of radiation therapy and the immune system is central for optimal tumor growth inhibition (4). The identification of rational approaches to design therapeutic strategies for the combination of radiation therapy with immunotherapy is still an unmet need. A better understanding of the molecular and cellular components of the emerging field of radio-oncoimmunology is central for the development of novel therapeutic approaches aiming at improving the effectiveness of radiotherapy.

In this review, we first highlight the diversity of cell death modalities elicited by ionizing radiation (IR) and focus on their immunogenic potentials. Next, we will briefly describe the roles of main innate immune cells in tumor microenvironment and then discuss the impacts of IR on various innate cells functions. We will also discuss how the modulation of innate immune cell functions by IR impacts on cancer treatment. A particular attention will be paid to dendritic cells (DCs), macrophages, natural killer (NK) cells, and myeloid-derived suppressor cells (MDSCs), since currently much more is known about these specific cell types.

## Ionizing Radiation Dictates the Death and the Immunogenicity of Cancer Cells

Despite the fact that radiation therapy plays a central role in cancer treatment, the biological processes that are involved in the effectiveness of radiotherapy are poorly understood. Even though various forms of cell death, including apoptosis, autophagic cell death, mitotic catastrophe, and cellular senescence, have been detected after IR (5, 6), the precise contribution of these lethal events to the biological effects of IR remains elusive.

### Ionizing Radiation Can Eliminate Cancer Cells through Distinct Cell Death Modalities

**Figure 1:**
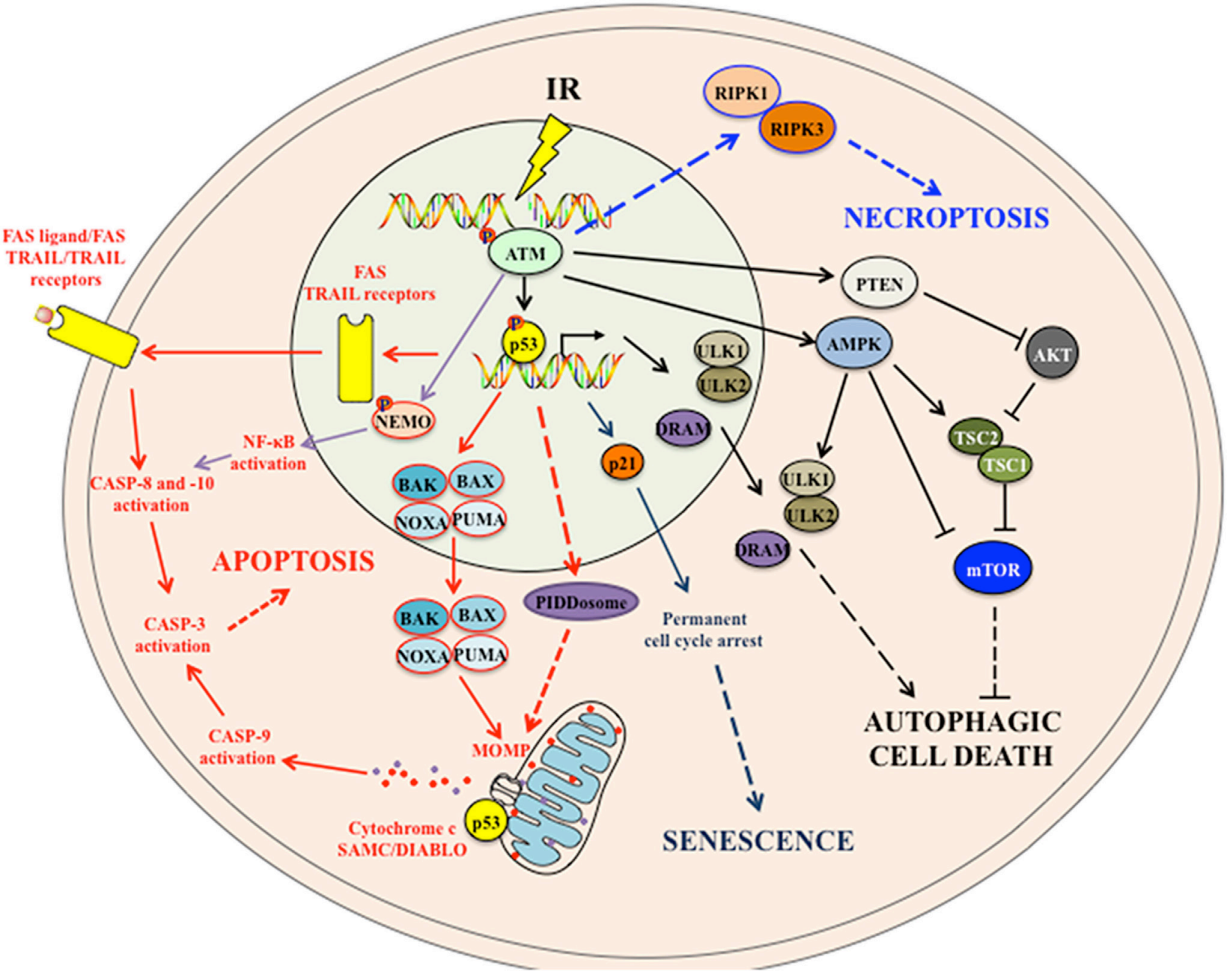
**The intracellular signaling pathways associated with IR-induced cell death modalities**. IR induces cellular apoptosis by activating both the intrinsic apoptotic pathway (through proapoptotic proteins-mitochondrial outer membrane permeabilization (MOMP)-Cytochrome *c*/SMAC/DIABLO release-caspase activation) and the extrinsic apoptotic pathway (through the upregulation of death receptors and the activation of downstream caspases). p53 also induces the expression of p53-inducible death domain (PIDD) protein in response to ionizing radiation, which acts as an effector of p53-dependent apoptosis. In addition, activated ATM following IR may activate the NF-κB pathway that in turn induces apoptosis. IR also leads to persistent DNA damages, which induce to p53 activation and p21 upregulation. p21 mediates cell cycle arrest and cellular senescence. Following IR, both activated ATM and p53 may trigger autophagic cell death to cells. ATM can activate AMPK and PTEN that suppress mTOR complex and induce autophagy. In addition, p53 upregulates the expression of autophagy-initiating kinase ULK1 and ULK2 and the damage-regulated autophagy modulator (DRAM) that subsequently induce autophagy. Note that it is still not certain whether this IR-induced autophagy would systematically lead to autophagic cell death. How IR induces necroptosis is still not fully understood. Some studies suggest that, in absence of caspase 8 activation, activated ATM following DNA damages (such as those induced by alkylating agent treatment) might mediate necroptosis by activating RIPK1 and RIPK3. See the main text for details. Abbreviations: AMPK, adenosine 5′-monophosphate (AMP)-activated protein kinase; ATM, ataxia-telangiectasia mutated; BAK, BCL-2 homologous antagonist/killer; BAX, BCL-2-associated X protein; IR, ionizing radiation; mTOR, mammalian target of rapamycin; NEMO, NF-kappa-B essential modulator; NF-κB, nuclear factor kappa B; PTEN, phosphatase and tensin homolog; PUMA, p53 upregulated modulator of apoptosis; RIPK, receptor-interacting protein kinase; TRAIL, TNF-related apoptosis-inducing ligand; TSC, tuberous sclerosis complex; ULK, UNC-51-like kinase.

After exposure to IR, cancer cells may die through distinct modalities (Figure 1). Apoptosis, autophagic cell death, necrosis, and necroptosis are cell death modalities that have been extensively studied and characterized. A nomenclature mainly based on morphological, biochemical, and enzymatic criteria has been proposed and ordered lethal processes in three types, with apoptosis as the type I cell death modality, the autophagic cell death as the type II cell death, and necrosis or necroptosis as type III cell modalities (7).

Apoptosis, which is the principal death modality detected after IR, is described as a programmed cell death (PCD) with specific morphological alterations such as the chromatin condensation (also known as pyknosis), the nuclear fragmentation (also known as karyorhexis), the plasma membrane blebbing, and the formation of apoptotic bodies that could be engulfed by phagocytes (7). Apoptosis can be triggered by two distinct interlinked signaling pathways, namely the intrinsic pathway driven by intracellular cues (such as DNA damage or metabolic alterations) and the extrinsic pathway driven by extracellular signals such as death ligands. In both pathways, apoptotic signals lead to the activation of initiator caspases (CASP) (such as CASP-9 for the intrinsic pathway and CASP-8 and -10 for the extrinsic pathway), through proteolytic cleavages. Once activated, these initiator proteases trigger a cascade of CASP activation by cleaving and activating downstream effector CASP (including CASP-3, -6, and -7). Consequently, the proteolytic processing of numerous cytoplasmic or nuclear substrates of CASP triggers the typical morphology of apoptotic cells. Initially associated with the induction of apoptosis, the biological activities of CASP may also participate to cellular processes that are independent of cell death modalities (such as macrophage activation or differentiation of skeletal myoblasts and keratinocytes) (8), indicating that the detection of the enzymatic activity of caspases in response to IR may not always be indicative of the execution of an apoptotic death.

Irradiated cells may also die through type a II cell death modality that is known as autophagic cell death (9). Initially, misnamed as autophagy (10), the autophagic cell death is a biological process distinct from autophagy. Autophagy is an evolutionarily conserved lysosomal pathway that participates in the maintenance of the cellular homeostasis by preventing the accumulation of misfolded and aggregated proteins as well as damaged organelles (11). This process, which starts with the nucleation of phagophore forms, produces, through lipid incorporation, the autophagosomes that will fuse with lysosomes to become autolysosomes that orchestrate the degradation of the sequestered content. This autophagic flux that is tightly regulated by autophagy-related (ATG) proteins (12) may either favor tumor growth by favoring the survival of cancer cells under unfavorable conditions (such as hypoxia and nutriment deprivation) or contribute to tumor suppression by triggering the death of cancer cells when they are resistant to apoptosis (13). The autophagic cell death is defined as a cell death process that occurs after the induction of autophagy and is blocked by inhibitors of autophagy function and/or genetic inactivation of autophagic modulators (14). The autophagic protein ATG5 was recently implicated in the induction of IR-induced autophagic cell death (15). This process is distinct from the induction of autophagy after IR where the inhibition of the mammalian target of Rapamycin (mTOR) or the kinase AKT increases cytotoxicity of IR (13), confirming that autophagy may also contribute to the resistance of cancer cells to IR. We recently revealed that autophagy may also be involved in the enhancement of radiation therapy effects in immune-competent mice (16), highlighting the fact that the autophagic machinery can contribute to the regulation of cancer cell fate during cancer treatment.

Necrosis and necroptosis are stereotypical forms of type III cell death modalities that are also detected after IR. Necrosis was initially described as an unordered cell death mode associated with an organelle swelling, the rupture of their plasma membrane and the cell lysis. This “accidental” death leads to the passive release of intracellular components such as adenosine triphosphate (ATP) or high mobility group box 1 (HMGB1) protein and causes an intense inflammatory response. Low doses of IR generally eliminate cancer cells through apoptosis, whereas high doses of IR can lead to necrosis (17). The characterization of the molecular mechanisms of necroptosis (18) revealed the ability of IR to induce a programmed necrosis in anaplastic thyroid and adrenocortical cancer cells (19). Necroptosis and necrosis share morphological characteristics (such as plasma membrane rupture, cell swelling, and the release of intracellular components to extracellular milieu), but in contrast to necrotic process, necroptosis is a regulated process that can be induced in response to death receptor activation or after apoptosis inhibition and regulated by receptor-interacting protein kinases 1 and 3 (RIPK-1 and -3) or mixed lineage kinase domain-like (18).

Ionizing radiation has also been associated with cell death modalities that do not or partially exhibit the morphological features, the biochemical alterations and the enzymatic activities above described. These less studied cell death processes have been defined as atypical cell death modalities. The mitotic catastrophe is one of these processes that can be induced after radiotherapy. In response to IR, tumor cells carrying mutated or inactivated p53 cannot efficiently activate cell cycle checkpoints (in particular G2/M checkpoint) to initiate cell cycle arrest and carry out DNA repair. Consequently, cancer cells containing unrepaired DNA enter prematurely into mitosis and undergo mitotic catastrophe (5). In addition, the irradiation of human keratinocytes with doses ranging from 0.005 to 0.5 Gy induces early apoptosis and necrosis with a substantial population of cells that undergo G2/M arrest and ultimately die through mitotic cell death (20), indicating that non-tumoral cells may also undergo a mitotic death after IR. Alternatively, mitotic catastrophe may result from the hyper-amplification of centrosomes as a result of failure to repair the DNA damages induced by IR, and lead to multipolar mitotic spindles and abnormal chromosomal segregation (21).

In addition to canonical cell death modalities, cellular senescence can also be induced in dose-dependent and cell type-dependent manners and contribute to the elimination of cancer cells after IR (22, 23). Cellular senescence is a state during which cells undergo irreversible growth arrest in response to various stimuli including oncogene or tumor suppressor gene activation, epigenetic disruption, oxidative stress, as well as DNA damage elicited by IR or several chemotherapeutic agents (24). This cellular process, which is activated and maintained by p53/p21- or p16^INK4a^/RB-dependent pathways, is considered as an antitumor barrier that halts the proliferation of cancer cells (24, 25). Senescent cells remain metabolically active and can secrete numerous proinflammatory cytokines, chemokines, growth factors, and proteases that collectively are known as senescence-associated secretory phenotype (SASP). Once released, SASP can act in an autocrine and/or paracrine manner to induce numerous either beneficial or noxious activities including induction of angiogenesis, modulation of cell proliferation and stem cell activity, stimulation of epithelial–mesenchymal transition, promotion of chronic inflammation, depending on the specific pathophysiological context (24). Thus, while cellular senescence represents a cell-autonomous tumor suppressor mechanism, radiation-induced senescence could impact on the neighboring cancer cells and favor tumor survival and growth.

### The Central Role of the Kinase Ataxia-Telangiectasia Mutated and the Tumor Suppressive Protein p53 in IR-Mediated Cell Killing

The kinase ataxia-telangiectasia mutated (ATM) and the tumor suppressive protein p53 play critical roles in coordinating DNA repair and cell fate determination when DNA damages are not repaired. Following sublethal doses of IR, DNA double-strand breaks are sensed by the MRE11-RAD50-NSB1 (MRN) complex, which in turn recruits and activates the apical kinases ATM mainly by favoring its autophosphorylation at serine 1981. ATM phosphorylates MRN complex, and other substrates including checkpoint kinase 2 (CHK2), p53-binding protein 1, and breast cancer gene 1 protein, which participate in sustaining DNA damage response signaling and in inducing S and G2/M arrest. ATM and CHK2 further phosphorylate p53, leading to its stabilization and activation of its transcription factor function. P53 upregulates the expression of p21 that induces the cell cycle arrest in G1. The initiation of DNA damage response by ATM and the induction of cell cycle arrest by p53 allow an efficient DNA repair process to restore genome integrity (26). However, when damages are not repaired efficiently, cell death programs are initiated.

#### The Kinase ATM Regulates Cell Death Modalities Elicited by IR

Upon IR-induced DNA DSBs, the kinase ATM and its downstream effector CHK2 kinase are phosphorylated and activate the tumor suppressive protein p53. The tumor suppressive protein p53 regulates through transcription-dependent or independent mechanisms the activation of both intrinsic and extrinsic apoptotic signaling pathways (27). Furthermore, the kinase ATM may also phosphorylate the NF-κB essential modulator (NEMO/IKK-γ) thus, leading to NF-κB activation and subsequent proapoptotic CASP-8 activation (28). The kinase ATM may also regulate autophagy and control the induction of cell death.

Although in some cases, the induction of autophagy via ATM-adenosine monophosphate-activated protein kinase (AMPK)-UNC-51-like kinase (ULK1) pathways was described to confer cytoprotective effect in Temozolomide-treated glioma cells (29), the regulation of autophagy through ATM-AMPK- tuberous sclerosis complex 2 (TSC2)–mediated suppression of mTORC1 by reactive nitrogen species lead to the loss of cell viability in breast cancer cells (30). In response to DNA damage induced by Topotecan, ATM phosphorylates phosphatase and tensin homolog and promotes its nuclear translocation and induces autophagy (31). Whether IR induces autophagy via similar signaling pathways should be further clarified. Instead, it is shown that ATM mediated IR-induced autophagy through activation of p38 mitogen-activated protein kinase (MAPK) and inhibition of mTOR pathway in human cervical cancer Hela cells. Pharmacological and genetic inactivation of ATM lead to decreased autophagy and hypersensitivity of Hela cells to IR (32). The role of ATM in IR-induced necroptosis has not been clearly demonstrated. ATM regulates alkylating DNA-damage agent-induced necroptosis through phosphorylation of histone protein H2AX (33). It is suggested that in response to DNA DSBs and in absence of CASP-8 activation, ATM might activate RIPK1 and RIPK3, which form necrosome and trigger necroptosis. However, this remains yet to be verified and clarified (28).

#### The Tumor Suppressive Protein p53 Contributes to IR-Induced Cell Death

The tumor suppressor p53 plays a center role in the regulation of numerous IR-induced cell death pathways. Following IR and DNA damages, the tumor suppressive protein 53 is phosphorylated at serine 15 and serine 20 by the kinases ATM and ATR and their downstream mediators CHK2 and CHK1. Once phosphorylated, p53 is dissociated from its negative regulator, the E3 ubiquitin ligase MDM2 and stabilized (34). Radiation can induce cell apoptosis via both intrinsic and extrinsic pathways. In the IR-induced intrinsic pathway, p53 induces the transcription of a number of proapoptotic proteins, including members of B-cell leukemia 2 (BCL-2) family such as the proapoptotic BCL-2-associated X protein (BAX) (35). Apart from its prominent role as a transcription factor, p53 also functions in the cytoplasm to induce apoptosis by directly activating the proapoptotic BAX and BAK (36). BH3-only proteins including p53 upregulated modulator of apoptosis (PUMA), NOXA and Bcl-2 interacting mediator of cell death (BIM) are also key initiators of apoptosis induced by IR (37–40). The protein p53 also induces the expression of p53-inducible death domain protein in response to IR, which acts as an effector of p53-dependent apoptosis (41). In addition, a number of antiapoptotic proteins are repressed, which further enhances IR-induced apoptosis. For instance, p53 negatively regulates *Bcl-2* gene expression (42). P53 also transcriptionally represses the expression of antiapoptotic *survivin* gene (43). Both activation of proapoptotic proteins and repression of ant-apoptotic proteins by IR subsequently lead to the formation of BAX-BAK pores in the mitochondrial outer membrane, triggering mitochondrial outer membrane permeabilization (MOMP). MOMP facilitates the release of toxic proteins such as cytochrome c and the proapoptotic SMAC/DIABLO into the cytosol, leading to the activation of the intrinsic apoptotic pathway by activating the initiator CASP-9 (28). IR triggers also extrinsic apoptotic pathways by upregulating death receptors. IR upregulates Fas expression in tumor cells in a wild type p53-dependent manner (44, 45). IR also induces the expression of the TNF-related apoptosis-inducing ligand (TRAIL) receptors Killer/DR5 (46, 47). Other TRAIL receptors including DCR1, DCR2 and DR4 can also be induced by IR and are regulated by the wild-type p53 (48). The upregulation of these death receptors by IR may facilitate extrinsic apoptosis. The death receptors assemble into a multiprotein complex called death-inducing signaling complex (DISC) which in turn serves as a scaffold for the recruitment and activation of the initiator CASP-8 and CASP-10, leading to the activation of extrinsic apoptosis pathway. In addition to the upregulation of death receptors, IR also generated ceramides via acid sphingomyelinase, which in turn acts on the mitochondrion or activates the proapoptotic stress-activated protein kinase/c-Jun N-terminal kinase pathway and initiates apoptosis (49, 50).

Like its pleiotropic roles in regulating IR-induced apoptosis, p53 also modulates autophagy at multiple levels in IR-exposed cells. The transcription factor p53 upregulates the expression of human autophagy-initiating kinase ULK1 and ULK2 and induces autophagy in response to DNA damage. This p53-regulated autophagy ultimately leads to DNA-damage-induced cell death. Interestingly, p53 also induces the expression of the damage-regulated autophagy modulator (DRAM), a lysosomal protein that induces autophagy, leading to p53-dependent apoptosis, linking autophagy to p53 and damage-induced apoptosis (51).

The cellular senescence induced by IR is mainly mediated by p53. Persistent DNA damage activates p53 that induces p21 expression and cell cycle arrest (24). It is also shown that reactive oxygen species (ROS) are essential for P53-mediated cellular senescence after IR (52). Alteration of p53-dependent activity affects IR-induced cellular senescence. For example, activation of P53 with Nutlin-3a sensitized lung cancer cells to IR through induction of premature senescence (53). The nerve injury-induced protein 1 (Ninjurin1, Ninj1) is a P53 target following IR that in turn suppresses the expression of P53. Accordingly, inactivation of Ninj1 suppresses cell proliferation but enhances P53-mediated apoptosis and cellular senescence (54).

## Ionizing Radiation of Tumor Cells also Favors the Development of Anticancer Immune Response

Apart from its direct genotoxic activity and tumor cell killing capacity, IR also enhances immune response via immunogenic properties of IR-induced cell death, upregulation of major histocompatibility complex (MHC) class I molecules and *de novo* tumor antigen production that collectively and coordinately prime and activate innate and adaptive immune systems to generate tumor-specific immune response.

### Ionizing Radiation Induces Immunogenic Cell Death

Immunogenic cell death (ICD) consists of a functionally peculiar type of apoptotic demise triggered by various specific stimuli that is able to activate an adaptive immune response against dead cell-associated antigens. ICD involves the emission of a series of immunostimulatory damage-associated molecular patterns (DAMPs) including cell surface exposure of endoplasmic reticulum chaperone calreticulin (CRT), secretion of ATP, and release of HMGB1 protein, occurring in a defined spatiotemporal sequence. These ICD-associated DAMPs bind to specific receptors, recruits antigen-presenting cells (APCs) that process and present the dead cell-associated antigens to CD8^+^ cytotoxic T cells. Activated adaptive immune responses mediate direct antitumor effects and may acquire a memory phenotype that contributes to long-term tumor control (55).

Ionizing radiation is shown to effectively promote tumor ICD (56). For example, in a mouse B16F10 melanoma model, irradiation of cutaneous tumor prior to resection is shown to induce a specific antitumor immune response and significantly reduces lung metastasis after systemic challenge with untreated melanoma cells. Radiation induces CRT exposure on melanoma cell surface leading to increased DC phagocytosis of tumor cells (57). Radiation also induces the secretion of ATP and HMBG1 in both dying and live tumor cells, leading to increased antigen-specific cytotoxic T lymphocytes (CTL)-mediated tumor cell lysis (58). The combination of IR and hyperthermia treatment on colorectal cells induces cell surface expression as well as extracellular release of the chaperon molecule heat shock protein 70 (HSP70). HSP70 is able to promote DC maturation as revealed by an upregulation of the co-stimulatory molecule CD80 and the chemokine receptor CCR7. In addition, this combined treatment enhances phagocytic activities of macrophages and DCs along with an augmentation of proinflammatory cytokines [such as interleukin (IL)-8 and IL-12] secretion (59). Importantly, radiation-induced ICD has also been observed in clinical settings. In patients with esophageal squamous cell carcinoma receiving chemo-radiation therapy, tumor antigen-specific T cell response and elevated serum HMGB1 are detected in 38% of patients. HMGB1, which is significantly upregulated in the chemoradiation-treated tumors, is associated with better survival (60).

### Ionizing Radiation Induces Tumor Antigen Expression

In addition, IR upregulates tumor associated-antigens and MHC class I complex that increase the recruitment of tumor antigen-specific T cells and activate T cell-mediated tumor killing (61–63). Early studies indicate that high-dose (from 25 to 100 Gy) gamma-irradiation induces the upregulation of the tumor rejection antigen (HSP gp96) on human cervical cancer cells that may increase immunogenicity of tumor cells (64). Other tumor-associated antigens such as carcinoembryonic antigen, colon-specific antigen, mucin-1 and MHC class I are upregulated by irradiation, which enhances antigen-specific T cell response (62, 65). Moreover, irradiation may also enhance FAS expression in tumor cells and sensitizes tumor cells to antigen-specific CTL killing via FAS/FAS ligand pathway. The combination of irradiation and CTL yields enhanced antitumor response (66). Therefore, irradiation may induce an “*in situ* vaccination” to improve antitumor immune response and also immunotherapy efficacy (61). These properties of IR are important as they contribute to the increased immunotherapy effects even in poorly immunogenic tumors (67).

### Ionizing Radiation Modulates Mutational Burden during Anticancer Treatment

In tumor cells, IR provokes massive DNA damages. However, a small part of tumor cells eventually develop resistance to IR-mediated killing and accumulate incorrectly repaired/unrepaired DNA damages. This adds to tumor mutational burden and might enhance tumor aggressiveness. On the other hand, IR-induced mutations might provide a pool of tumor neoantigens that can be recognized and targeted by immune system (68). Indeed, it is shown that IR induces novel peptide synthesis in tumor cells and enhances antigen presentation by MHC class I molecules (63). Consequently, the specific expression of tumor neoantigens driven by tumor-specific mutations could be used as biomarkers of radiation therapy efficacy and could contribute to the development of novel therapeutic approaches (69).

## The Tumor Microenvironment Irradiation Dictates Antitumor Innate Immune Response

Tumors are composed of tumor cells and tumor stroma. Tumor stroma contains cellular components (such as fibroblasts, endothelial cells, myeloid-derived cells, and lymphocytes), vascular and lymphatic vessels, non-cellular supporting structures, cytokine, and chemokine milieu. Innate immune cells such as DCs (DCs), macrophages, natural killer (NK) cells, neutrophils, and other myeloid-derived cells such as MDSCs have been found in various tumors (70).

Tumor-infiltrating DCs are found in many different types of cancers and are reported to be associated with both good and poor prognosis depending on the types of studied tumor. Although DCs represent the most important APCs to cross-present tumor antigens to effector T cells and to activate antitumor T cell response, these essential capacities are paralyzed by tumor-derived inhibitory factors including IL-10, TGF-β, vascular endothelial growth factor A (VEGF-A), and arginase (71). In many cases, tumor-infiltrating DCs gradually develop an immunosuppressive phenotype characterized by lower expression of co-stimulatory molecules, decreased antigen-presenting activity and upregulation of regulatory molecules and receptors such as PD-1 and TIM-3 within tumor-microenvironment, as the tumor grow from early stages to advanced diseases (71, 72). Thus, restoring immunostimulatory capacities of tumor-infiltrating DCs and administration of antigen-loaded autologous DC vaccines may have important implications in the development of more efficient antitumor therapies (73, 74).

Tumor-infiltrating macrophages or tumor-associated macrophages (TAMs) are the major myeloid cells found in the tumor area. TAMs are derived from peripheral blood monocytes and are recruited to the tumor area by various tumor-derived chemokines and cytokines such as colony stimulating factor-1 (CSF-1), C-C motif chemokine ligand 2 (CCL2), stromal cell-derived factor-1 (SDF-1), and VEGF-A. Other factors such as hypoxia and tumor cell metabolites also contribute to TAMs infiltration. TAMs are differentiated and skewed toward protumorigenic phenotype within distinct tumor microenvironment such as hypoxia, acidity, and immunosuppressive cytokine milieu (75). TAMs contribute to tumor growth, angiogenesis, invasiveness, and metastasis. TAMs also express high level of ligands for PD-1 and CTLA-4 that exert immunosuppressive functions on T cells. In addition TAMs interfere with T cells activation by depleting L-arginine in the milieu that is important for T cell receptor ζ chain expression. Other inhibitory mechanisms include induction of T cell apoptosis and production of anti-inflammatory cytokines such as IL-10 and TGF-β. In addition, TAMs induce the recruitment of immunosuppressive regulatory T cells through the expression of chemokines such CCL5, CCL20, and CCL22 (76). Thus, TAMs infiltration was associated with poor clinical outcomes in the majority of cancers (77). Reversing these adversary roles of TAMs will be important in improving anticancer therapy efficacies.

NK cells also play important roles in antitumor immunity. This is not only due to their direct tumor cell-killing function via granzyme B/perforin pathway and other death-receptor pathways, but also due to their ability to secrete a plethora of proinflammatory cytokines and chemokines that regulate and promote innate and adaptive immune response (78). However, as in the cases of DCs and macrophages, cytotoxic functions of NK cells are often impaired within tumor microenvironment. Various factors including cytokines and tumor metabolites directly inhibit maturation, proliferation, and functions of NK cells. In addition, other tumor-infiltrating cells such as MDSCs, TAMs, and regulatory T cells also inhibit the functions of NK cells (78). Accordingly, several NK cell-based *in vivo* approaches including the activation of NK cells with stimulatory cytokines, the induction of antibody-dependent cell-mediated cytotoxicity and IFN-γ production with tumor antigen-specific monoclonal antibodies, and the enhancement of the cytolytic activity of NK cells with blocking antibodies against inhibitory signals, may increase the chances for successful cancer treatment (79).

Myeloid-derived suppressor cells are a group of heterogeneous immature myeloid cells with suppressive activities on both innate and adaptive immunity. MDSCs differentiate from common myeloid progenitors and are often composed of cells at varied differentiation stages. MDSCs may be grouped into monocytic MDSCs and granulocytic MDSCs. Tumor-derived cytokines and growth factors such as VEGF, IL-6, granulocyte CSF, granulocyte-macrophage CSF, and other proinflammatory mediators such as IL-1β, IL-17, HMGB1, cyclooxygenase 2 (COX_2_), and prostaglandin E2 (PGE_2_) induce MDSCs accumulation, differentiation, proliferation, and acquisition of immunosuppressive functions (80, 81). MDSCs exert their immunosuppressive roles on T cells through multiple mechanisms, including secretion of anti-inflammatory IL-10 and transforming growth factor-β (TGF-β) that inhibit functions of T cells and NK cells, generation of ROS and nitric oxide (NO) that interfere with T cell proliferation and activation, and interaction with other immune cells such as TAMs that together create a protumorigenic microenvironment (80). Like TAMs, MDSCs express high levels of PD-L1 that induces T cell exhaustion and arginase I that depletes l-arginine that is essential for T cell activation. MDSCs induce also regulatory T cell accumulation and impair NK cell cytotoxicity (80). Therefore, MDSCs are prominent players that can support tumor growth and inhibit antitumor immunity and thus represent another major obstacle to overcome for effective antitumor therapies.

Other tumor-infiltrating innate immune cell such as neutrophils, Langerhans cells, and eosinophils that have emerged as potential players in tumor development are also promising targets to improve the efficacy of cancer treatment (82–85). For example, tumor-associated eosinophils have been revealed to play essential roles in orchestrating effective antitumor response. Eosinophils were shown to produce chemo-attractants that recruit effector T cells into the tumor. Eosinophils induce also macrophage activation and tumor vascular normalization that together contribute to tumor suppression (85). Currently, the role of eosinophils in tumor immunity is under more in depth investigation and the impact of radiation therapy on the functions of tumor-associated eosinophils remains largely unknown.

### Ionizing Radiation Modifies Innate Immune Cell Migration and Homing

Tumor irradiation facilitates tumor antigen capturing and enhances tumor antigen presentation by DCs (86). Irradiation down regulates DC chemoattractant CCL21 expression in tumor tissue, which reduces the retention of DCs in tumor area after irradiation (86). On the other hand, irradiation also upregulates the expression of CCL21 on lymphatic vessels (87). These together may facilitate DCs homing to lymph nodes. These effects promote the ability of DCs to cross-prime and activate T cells (86). In contrast, another study demonstrated that gamma-irradiation (2Gy-8Gy) inhibited the migration murine DCs both *in vitro* and *in vivo*, in part due to a decreased expression of CCR7 and an increased apoptosis induced by irradiation in DCs (88).

Similarly, IR impacts profoundly on macrophage migration. A total of 10 Gy cranial γ-irradiation induces the expression of inflammatory mediators that serve as chemoattractant to promote the influx of peripheral blood-derived CCR2^+^ macrophages into the mouse brain (89). In the context of tumors, IR also induces macrophage recruitment. Tumor hypoxia due to a radiation-induced disruption of tumor vessels creates a transient hypoxic microenvironment and increases the expression of tumor-derived CSF-1, SDF-1 that together induces recruitment as well as anti-inflammatory activation of TAMs after radiation therapy (90–92). In addition, IR upregulates M-CSF expression by pancreatic ductal adenocarcinoma cells, which induces macrophage recruitment and differentiation toward M2-like phenotype (93). Of note, clinical studies also revealed that radiation therapy induced CSF-1 augmentation as well as the protumoral activation of macrophages, which were both associated with an impaired radiation therapy efficacy in prostate cancer (94). Combined radiation therapy with a anti-CSF-1 antibody or CSF-1R inhibitor treatment showed an improved antitumor effect (95) and will be significant to be further evaluated in clinical trials. Another important monocyte-chemoattractant CCL2 is also upregulated by IR and mediates macrophage recruitment into non-small cell lung cancer (96).

Irradiation-induced apoptosis increased neutrophils infiltration to the thymus (97). These recruited neutrophils were important in thymus regeneration after whole-body X-irradiation through their expression of SDF-1 (98, 99). Further characterization of the neutrophil infiltrating the tumors and the functional impact of irradiation on tumor-associated neutrophils should help for the development of novel therapeutic strategies.

Single high-dose (30 Gy) irradiation of the skin induced significant accumulation of eosinophils and the production of eosinophil-related cytokines such as IL4, IL-5, IL-13, IL-33, and CCL11 (100). A recent study showed that although synchroton microbean radiation treatment did not induce a significant difference in eosinophils infiltration pattern in murine mammary tumors as compared to synchroton broad-beam treatment, they did differentially regulate a subset of genes (*Ear11, Ccl24, Ccl6, Ccl9*) that were related to eosinophil functions and recruitment (101).

### Phagocytosis and Antigen Presentation Are Altered after IR

The effect of *in vitro* direct irradiation on DCs depends on irradiation doses and DCs maturation states. For example, 5 Gy gamma-irradiation downregulated the expression of costimulatory receptors CD80/CD86 on immature monocyte-derived DCs but did not affect these receptors on mature DCs or their ability to stimulate autologous T cells (102). Another study showed that when irradiated at 30 Gy, CD86 expression was increased on immature DCs and decreased on mature DCs, while other markers remained unaffected by irradiation. However, in this study, irradiation impaired the stimulatory effects of both immature and mature DCs on the proliferation of allogenic T cells (103). Irradiation also affected DCs functions differentially in that it inhibited DCs response to endogenous antigens but enhanced DCs response to exogenous antigens (104). The divergent effects of irradiation on DCs were not due to defect in maturation or in presenting endogenous antigens, but were rather a result of the inhibition of proteasome function by irradiation. This in part accounted for the decreased endogenous antigen processing and possibly enhanced MHC class I molecules recycling and exogenous antigen presentation. Accordingly, irradiation abrogated DCs-induced endogenous antigen-specific T cell response and tumor suppression. On the contrary, irradiation enhanced the ability of DCs to activate T cell response to exogenous antigens and inhibited the growth of exogenous antigen-expressing tumors (104). Therefore, different irradiation doses, DCs maturation states and different types of antigens influence the outcomes of DCs activation following direct irradiation.

Like DCs, Langerhans cells residing in the skin and mucosa are endowed with potent antigen-presenting capacities at the first line of immune defense (105). An early study examining the prognostic role of Langerhans cell infiltration in uterine cervical squamous cell carcinoma patients treated with radiation therapy, showed that Langerhans cell infiltration was significantly associated with higher 5-year overall survival, suggesting that Langerhans cell infiltration after radiation therapy might mediate the immune response through their antigen presenting capacity and enhance the antitumor effect (106). Indeed, it was demonstrated that Langerhans cell infiltration after radiation therapy was associated with increased T cell infiltration and with improved local tumor control in cervical cancer (107, 108). However, in other settings, Langerhans cells may also limit the effect of radiation therapy. Epidermal Langerhans cells are more radioresistant than dermal DCs due to an overexpression of p21 and the capacity of the rapid repair of DNA damages induced by irradiation. Following radiation, Langerhans cells migrate to skin-draining lymph nodes in a CCR7-dependent manner. It is shown that Langerhans cell induced immunosuppressive regulatory T cell accumulation in the tumor is in part due to an upregulation of MHC class II expression on migratory Langerhans cells after irradiation. Consequently, Treg cells accumulation mediates immune suppression and tumor resistance to radiation therapy (105). Therefore, it appears that the *in vivo* impacts of IR on Langerhans cells might depend on the tumor types as well as the induction of different types of T cell infiltration (effector T cells or regulatory T cells).

### The Differentiation and the Activation of Innate Immune Cells Is Modulated by IR

Radiation induces tumor cells death that leads to the release of tumor antigens, HSPs and other danger signals. These products then stimulate DC maturation. Although some *in vitro* studies arguing that IR compromises the stimulatory activities of DCs, *in vivo* models demonstrate that IR enhances the ability of DCs to capture tumor antigens (86) and promotes DC migration to draining lymph nodes in a way that is dependent on toll-like receptor signaling pathway, where they present tumor antigens to T cells and induce antigen-specific T cell response (109).

Various factors determine the impacts of IR on macrophage functions. One prominent factor is irradiation doses. For example, it was reported in many studies that low-dose (≤1 Gy) irradiation inhibited the proinflammatory activation of macrophages (110). Low-dose irradiation also inhibited oxidative burst in activated macrophages (111). On the contrary, high-dose (≥1 Gy) irradiation tends to induce a proinflammatory phenotype on macrophages with increased production of proinflammatory cytokines such as IL-1β and expression of induced nitric oxide synthase (iNOS) (112–114). Another important factor lies in macrophages. Macrophages from different mouse strains show variant intrinsic radiosensitivity. For example, irradiation enhanced anti-inflammatory characteristics of macrophages from C57BL/6 mice that are supposed to be more radioresistant, whereas macrophages from CBA/Ca mice that are more radiosensitive retain a proinflammatory feature after irradiation (115). Irradiation also differentially affected functions of macrophages from BALB/c and C57BL/6 mice (116).

In the tumor context, to date IR has been shown to either enhance the protumorigenic properties of TAMs or reprogram them toward antitumoral phenotypes in different experimental settings. For examples, IR induces M2-like protumorigenic TAMs that contribute to tumor recurrence and treatment failure. This is due to CSF-1 expression in murine prostate tumor cells that induced the recruitment of TAMs and MDSCs. Combined treatment with irradiation and CSF-1R inhibitor markedly improved antitumor efficacy (94). Macrophages from irradiated tumors show increased expression of arginase 1 (Arg1), COX_2_, and iNOS that promote tumor growth (117, 118). Macrophages also increased the expression of VEGF that led to tumor neovasculogenesis (119). However, there were also studies showing that radiation therapy could redirect TAMs from protumorigenic to antitumoral cells. For example, low-dose (2 Gy) whole-body irradiation induced iNOS expression and the production of proinflammatory cytokines such as tumor necrosis factor-alpha (TNF-α), IL-12 (p70), and IFN-γ in peritoneal macrophages and TAMs (120). A recent study on murine insulinoma demonstrated that low-dose (2 Gy) irradiation induced iNOS expression in macrophages both *in vitro* and *in vivo*. This reprograming of proinflammatory macrophages by irradiation led to tumor vascular normalization and increased the effect of T cell immunotherapy (121). Furthermore, irradiation combined with 2-deoxy-d-glucose or hyperthermia also activated macrophages toward proinflammatory phenotype (122). These results suggest that depending on studied tumor models and the specificity of the used treatment regimen, irradiation may have different effects on TAMs functions that can in turn impact on tumor response and treatment outcomes.

The roles of neutrophils in tumor immunobiology are just emerging and little is known at the moment about the impact of IR on tumor-associated neutrophils. For instance, low-dose (0.512 Gy) irradiation suppressed myeloperoxidase activity and reactive nitrogen species generation in neutrophils from guinea pig (123). On the other hand, high-dose (20 Gy) irradiation induced oxygen free radicals in rat neutrophils (124). However, the effects of irradiation on human neutrophils are less known.

### Ionizing Radiation Changes Cytokine Secretion Profiles

Different doses of irradiation yield different functional modulations to DCs. Low-dose irradiation seems to have divergent effects on DCs in many reports, possibly due to different experiment designs. For instance, low-dose at 0.05 Gy of gamma-irradiation of murine DCs significantly induced IL-2, IL-12, and interferon-γ (IFN-γ) production in DCs that promote T cells proliferation (125). At a dose of 0.2 Gy, gamma irradiation increases the surface expression of CD80, CD86, MHC class I and II molecules in murine DCs but inhibits their capacity of antigen uptake. In addition, this low-dose irradiation suppresses IL-12 production in DCs, but increases IL-10 production, implying a shift to immune tolerance (126). However, low-dose irradiation (from 0.05 to 1.0 Gy) did not affect surface markers or cytokine production in neither immature nor mature human DCs, and had no influence on the capacity of DCs to stimulate T cell proliferation (127), suggesting that the impact of low-dose irradiation on DCs function might be different from mouse to human.

High dose of irradiation also impacts on DCs differently. Irradiation at 30 Gy did not impact on DCs endocytic, phagocytic and migratory capacity but significantly inhibited IL-12 production by mature DCs while IL-10 production was unaffected (103). Inhibition of IL-12 expression in DCs by irradiation was in part mediated by an increase of IL-6 and activation of down stream signal transducer and activator of transcription 3, which led to inhibition of c-REL transcription factor (128). In addition, irradiated peptide-pulsed mature DCs showed impaired ability to prime naïve CTL (103). Likewise, gamma-irradiated (30 Gy) DCs derived from peripheral blood mononuclear cell of multiple sclerosis patients showed significantly reduced surface expression of costimulatory CD86 and had lower capacity to promote T cell proliferation as compared to non-irradiated DCs. These irradiated DCs also upregulated IL-2 and IL-4 secretion by T cells (129). Although high-dose irradiation might directly inhibit functions of DCs, another study showed that irradiation (3 × 5 Gy) induced tumor cell death that triggers DC maturation and production of proinflammatory cytokines such as IL-6, IL-8, IL-12p70, and TNF-α (130). Irradiation from 10 to 60 Gy also upregulates CD70 expression on mature DCs, an event that is correlated with the ability of these cells to stimulate T cell proliferation and IFN-γ production (131).

Although, in many *in vitro* studies, irradiation was shown to inhibit the antigen presentation capacity and the production of proinflammatory cytokines in DCs, *in vivo* studies seems to reflect opposite effects, possibly due to the complexity of the microenvironment that cooperatively influences the maturation and the activation of DCs. It might also be possible that combined direct and indirect effects of *in vivo* irradiation promote distinct DC functions in a context that significantly differed from *in vitro* irradiations. For example, although X-ray irradiation at 6 Gy significantly suppressed IL-23 secretion and slightly inhibited IL-12p70 production in DCs, irradiated fibroblast still interacted with and stimulated DCs to maintain IL-23/Th17 response (132). Thus, direct and indirect impacts of high-dose irradiation on DC activation could be quite different even opposite. This may explain why in many preclinical models, additive or synergic effects of DCs administration and radiation therapy were often documented.

As mentioned above, IR can directly modulate macrophage activation phenotype and their cytokine expression profiles. In addition, IR impacts on macrophage functions indirectly through the interaction of IR-induced cell death with macrophages. Irradiation-induced tumor cell death, in particular apoptosis, has previously been regarded as non-immunogenic (133). Apoptotic cells induced the secretion of anti-inflammatory cytokine IL-10 in macrophages (134). However, accumulating studies have also pointed out that apoptosis triggered by a subset of antitumor treatments may have immunogenic effects (133, 135). In addition, while the engulfment of apoptotic cells by non-stimulated or M2 macrophages induced the expression of anti-inflammatory macrophage markers such as TGF-β, such engulfment by M1 macrophages enhanced proinflammatory properties as indicated by an increased production of iNOS, superoxide, IL-6, and TNF-α (136). ICD induced by irradiation leads to the release of HMGB1 and the secretion of ATP (56). Upon ligation with TLR4, HMGB1 triggers NF-κB activation (137). ATP binds to P2X7 purinergic receptor and activates the NLRP3 inflammasome (138). NF-κB and NLRP3 inflammasome activation are both involved in the expression and maturation of proinflammatory cytokines such as IL-1β (139).

### Innate Immune Cell-Mediated Cytotoxicity Is Affected by IR

Interestingly, apart from the enhancement of antigen-presenting capacity of DCs, irradiated tumor cells can induce the expression of granzyme B and perforin in DCs and directly stimulate DCs cytotoxicity to kill tumor cells (140). Although gamma-irradiation induces DCs accumulation in the tumor area that further activates tumor-specific T cell (141), it is noteworthy that radiation therapy induced upregulation of tumor antigens may also confer suppressive effects on DCs. For example, radiation-induced breast tumor-derived gamma-synuclein was shown to inhibit the expression of costimulatory molecules CD40 and CD86, and decrease the expression of proinflammatory cytokines in DCs. Gamma-synuclein-treated DCs also inhibit T cell proliferation but induce TGF-β production in T cells and increase the population of immunosuppressive regulatory T cells (142).

It was also demonstrated that in irradiated tumors, while the expression of costimulatory molecules is upregulated, the expression of PD-L1 and PD-L2 on DCs (140), which are known to inhibit antitumor immunity (143), are significantly reduced. Contradictorily, some other studies show that IR upregulate the expression of PD-L1 on tumor cells, DCs and TAMs that limit the antitumor effect of radiotherapy. The combined therapy of irradiation and anti-PD-L1 treatment resulted in activation of cytotoxic T cells and synergistic elimination of MDSCs by T cell-generated TNF, which is associated with delayed tumor growth (4, 144).

Irradiation can directly affect NK cell functions. *In vitro* studies showed that X-ray irradiation at 5 to 15 Gy could transiently increase human NK cell activity to lyse tumor cells that could be maintained in the presence of interferon (145). It was reported that the cytotoxic activity of human peripheral blood NK cells augmented following an irradiation dose at 1 Gy that peaked at 6 Gy and then decreased gradually when irradiation dose reached 16 Gy. Similarly other studies showed that human NK cells activity was enhanced following irradiation at 5–20 Gy (146, 147). In addition, low-dose gamma irradiation at ≤0.2 Gy induced expansion of NK cells, augmented NK cell cytotoxicity (148) and the expression of Fas ligands and perforin, and significantly increased the expression of IFN-γ and TNF-α in NK cells in a p38MAPK-dependent manner (149). Irradiation can also affect NK cell functions through the modulation of interaction between tumor cells and NK cells. For example, irradiation upregulated the expression of natural-killer group 2, member D (NKG2D) ligand and HSP70 in tumor cells that may increase susceptibilities of tumor cells to NK cell-mediated cytolytic attack (150, 151). Combined treatment of radiation therapy and histone deacetylase inhibitor was shown to increase the expression of NKG2D ligand expression and enhance the susceptibilities of lung cancer cells to NK cell cytotoxic activities (152). IR also triggers the release of second mitochondria-derived activator of caspase (Smac) from mitochondria that competes with X-linked inhibitor of apoptosis protein and enhances NK cell-mediated apoptosis of tumor cells (153).

### Ionizing Radiation May Also Trigger the Elimination of Innate Immune Cells

Radiation therapy is a prominent source of myelosuppression during cancer treatment, especially when combined with chemotherapy. This is in particular the case when radiation therapy is delivered to pelvis such as for cervical cancer, rectal cancer and prostate cancer, during which a large proportion of bone marrow is affected (154). Neutrophils are the major innate immune cells that are decreased by radiation therapy. Up to 90 and 80% of cervical cancer patients underwent a grade II or worse neutropenia during 3D conformal radiotherapy and intense-modulated radiation therapy, respectively (155).

Myeloid-derived suppressor cells have been shown to accumulate in many cancer patients. In hepatocellular carcinoma, the basal level of CD14^+^HLA^−^DR^−/low^ MDSCs is higher than that in healthy controls. Radiotherapy significantly reduced the frequency of CD14^+^HLA^−^DR^−/low^ MDSCs that was negatively correlated to patient overall survival, indicating that a reduction of MDSCs after radiotherapy could be used as a prognostic factor in hepatocellular carcinoma patients (156). Radiation therapy of tumors also leads to a decrease of peripheral MDSCs that re-expand upon tumor recurrence. Declined MDSCs population was associated with increased T cells proliferation and T cells response to tumor-associated antigens (157). In patients with oligometastases, stereotactic body radiotherapy (SBRT) when combined with the multitargeted tyrosine kinase inhibitor Sunitinib, induced a decrease of peripheral blood CD33^+^CD14^+^CD16^+^ monocytic MDSCs as well as Tregs and B cells, along with an increase of Tbet expression in primary CD4^+^ and CD8^+^ T cells, which was associated with improved progression-free survival. A reduction of monocytic MDSC in this setting thus may be considered a valuable biomarker for predicting clinical outcomes (158).

Early studies have shown that gamma-ray or X-ray irradiation also decreases the number of epidermal Langerhans cell in human skin (159, 160). Similarly, in a dose-dependent manner, irradiation depleted mouse epidermal Langerhans cells population that was recovered after the stop of irradiation (161–163).

Effective DNA damage sensing followed by efficient and faithful DNA repair to restore genome integrity is vital for cell functions and cell survival, as reflected by the fact that germline mutation of *ATM* and *TP53* caused hereditary defects in DNA damage signaling and repair pathway lead to predisposition of cancer and many other diseases such as immune deficiency (164).

Dysfunction in ATM (murine analog of human ATM) results in the accumulation of unrepaired DNA in the cytoplasm upon DNA damage. These free DNA fragments are sensed by STING (stimulator of interferon genes)-mediated pathway, which activates the expression of Toll-like receptors (TLRs), RIG-I-like receptors and promotes induction of type I interferons, leading to enhanced antiviral and antibacterial response in *Atm^−/−^* mice (165). DNA DSBs also activate the transcription factor interferon regulatory factor 3 (IRF-3) in a manner dependent on ATM-IKKα/β, leading to cell-autonomous production of interferon β (166). Further, persistent ROS are shown to induce chronic activation of ATM that triggers a continuous activation of NF-κB pathways, contributing to aggressive phenotype of cancer cells (167). Indeed, ATM has been shown to regulate NF-κB activity by mediating nuclear NEMO SUMOylation and subsequent ubiquitination, an event that leads to NEMO relocation to the cytoplasm and NF-κB activation through the canonical pathway (168).

P53 was recently demonstrated to participate in the regulation of macrophages functions. P53 is involved in the proinflammatory macrophage activation and in addition, P53 suppresses the anti-inflammation phenotype of macrophages (15). P53 cooperates with NF-κB to induce proinflammatory genes expression in macrophages (169). P53 may directly activate IRF-5 (170), a dominant transcription factor in proinflammatory macrophage activation (171).

## Concluding Remarks

**Figure 2:**
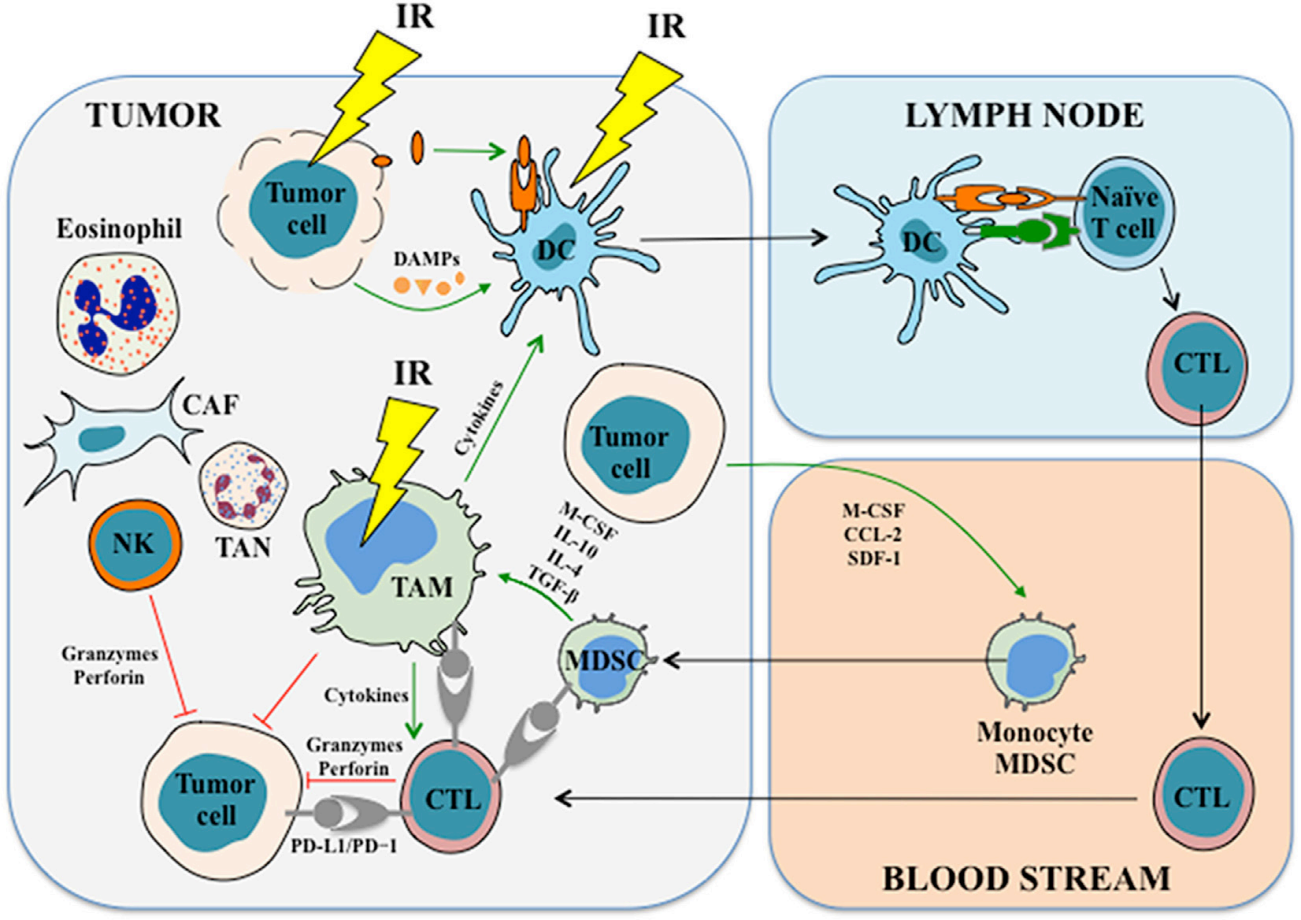
**The effects of IR on immune cells**. IR induces immunogenic cell death in tumor cells, leading to the release of tumor antigens and damage-associated molecular patterns (DAMPs), which in turn prime and activate antigen-presenting cells (APCs) such as dendritic cells. APCs stimulate and activate T cells in the lymph nodes and lead to the generation as well as the proliferation of tumor antigen-specific cytotoxic T cells (CTLs), which then migrate into the tumor to exert antitumor functions and mount the antitumor immune response. IR also has profound impacts on tumor-associated macrophages (TAMs). For example, IR induces macrophage infiltration and differentiation in the tumor. In some cases, IR promotes proinflammatory macrophage activation and enhances their immunostimulatory and tumoricidal activities. In addition, accumulating studies revealed that IR might modulate functions of other innate immune cells, such as myeloid-derived suppressor cells, NK cells, tumor-associated neutrophils, and probably other types of cells. See the main text for details. Abbreviations: CAF, cancer-associated fibroblast; CCL-2, chemokine (C–C motif) ligand-2; CTL, cytotoxic T lymphocyte; DC, dendritic cell; IL, interleukin; IR, ionizing radiation; M-CSF, macrophage colony-stimulating factor; MDSC, myeloid-derived suppressor cell; NK cell, natural killer cell; PD-1, programmed cell death protein 1; PD-L1, programmed cell death ligand 1; SDF-1, stromal cell-derived factor-1; TAN, tumor-associated neutrophil; TGF-β, transforming growth factor β.

While interventions aiming at improving the efficacy of IR by the combination T cell directed approaches (such as PD-1/PD-L1 blockades) and IR are growing in the clinic, there is mounting evidence that IR also primes and induces the activation of an adaptive antitumor immunity through the induction of ICD, the release of tumor antigen, the stimulation of inflammatory response, and the modulation of immune cell functions, which can facilitate and enhance immunotherapy effects and potentially reduce immunotherapy-related adverse events (Figure 2). However, the impact of radiation on innate immune cells may be tumor type dependent and vary in relation with the specificity of the used treatment protocol. On the other hand, many reports indicate that in certain cases radiation therapy creates a more immunosuppressive microenvironment due to the upregulation of PD-L1, a transient potentiation of tumor hypoxia, or an alternative activation of TAMs, indicating that the addition of immunotherapy to the treatment protocol can overcome these obstacles, increase radiosensitivity and may lead to an enhanced systemic effect of radiation therapy. For these reasons, there is a strong rationality for combining radiation with immunotherapy for cancer treatment. A deeper understanding of the molecular mechanisms that are involved in the modulation of innate immune cell functions, particularly in the context of tumor microenvironment, is thus fundamental for the development of new therapeutic strategies targeting the inhibitory effects of tumor-infiltrating cells and for the restoration of their antitumor activities.

## Author Contributions

QW, AA, IM, CB, NM, ED, and JLP provided advices and wrote the article. QW designed and produced the figures. JLP edited the paper.

## Conflict of Interest Statement

The authors declare that the research was conducted in the absence of any commercial or financial relationships that could be construed as a potential conflict of interest.
